# Lymphatic filariasis among the Yakurr people of Cross River State, Nigeria

**DOI:** 10.1186/1756-3305-5-203

**Published:** 2012-09-19

**Authors:** Cletus I Iboh, Okpok E Okon, Kenneth N Opara, Joseph E Asor, Susan E Etim

**Affiliations:** 1 Department of Biological Sciences, Cross River University of Technology, Calabar, Nigeria; 2 Department of Zoology & Environmental Biology, University of Calabar, Calabar, Nigeria; 3 Department of Zoology, University of Uyo, P. M. B. 1017, Uyo, Akwa Ibom State, Nigeria

**Keywords:** Prevalence, *Wuchereria bancrofti*, Yakurr, Cross river, Nigeria

## Abstract

**Background:**

In order to initiate a disease elimination programme for lymphatic filariasis based on mass drug administration, a proper understanding of the geographical distribution and degree of risk is essential.

**Methods:**

An investigation of lymphatic filariasis due to *Wuchereria bancrofti* was carried out among 785 people in four communities of Yakurr Local Government Area of Cross River State, Nigeria between March and August, 2009. Finger prick blood smear samples collected from the subjects were examined for *W. bancrofti* using standard parasitological protocol. The subjects were also screened for clinical manifestations of lymphatic filariasis.

**Results:**

Of the 785 persons examined, 48 (6.1%) were positive for microfilariae in their thick blood smear. There was a significant difference in the prevalence of lymphatic filariasis among the various age groups (P < 0.01) although peak prevalence occurred between 41 – 60 years. There was no significant difference in prevalence and density with respect to sex (P > 0.05). The overall mean microfilarial density of the infected individuals was 5.6mf/50 μl. There was a significant variation (P < 0.01) in mean microfilarial density within the communities, ranging from 4.7 to 6.4 mf/50 μl. The only clinical sign found in the study area was lymphoedema of the leg recording 2 (0.3%) prevalence.

**Conclusions:**

The National Lymphatic Filariasis Elimination Programme should intervene by expanding the distribution of albendazole and ivermectin to all endemic areas including Yakurr Local Government Area of Cross River State, Nigeria.

## Background

Lymphatic filariasis is a major public health problem, affecting 120 million people living in 72 countries of the world [1], 39 African countries carry over a third of the global burden of lymphatic filariasis [1]. Lymphatic filariasis is associated with dermatitis, elephantiasis and hydrocoele [2]. Severe complications could include lymphoedema and elephantiasis of the limbs or genitalia, which adversely affect personal and social life, and limit occupational activities.

Filariasis is one of the most common causes of permanent disability worldwide creating the highest disease burden in terms of DALYs among tropical disease [3]. The disease is a major cause of poverty as it leads to economic burden for those affected, their dependants, their communities and their country as a whole [4,5]. Consequently, in 1998, the Global Alliance to Eliminate Lymphatic Filariasis (GAELF) was formed to support the lymphatic elimination programme in endemic countries and The World Health Organization launched a Global Programme to Eliminate Lymphatic Filariasis (GPELF) [6], which has the goal of eliminating the disease as a public health problem by 2020. The strategy aimed at achieving this goal is twofold. First, interrupt transmission of the LF parasite by delivering single annual doses of diethylcarbamazine (DEC) or ivermectin plus albendazole to the entire eligible population living in areas where the disease is endemic (defined as areas where the prevalence of microfilaraemia or antigenaemia is ≥1%). In addition to interrupting transmission, mass drug administration (MDA) provides significant collateral health benefits, such as reduced morbidity from intestinal worms and ectoparasites (for example, lice). Second, alleviate suffering and disability by introducing basic measures, such as improved hygiene and skin care, for those with lymphoedema and by providing surgery for men with hydrocele [7].

Available literature on the disease from both the North and Central parts of Nigeria and the report of a postal survey by the Nigerian Lymphatic Filariasis Elimination Programme (NLFEP) have shown that lymphatic filariasis is endemic [8-10]. One of the greatest challenges confronting the NLFEP is the lack of information on the distribution and degree of risk of the disease in the country.

The NLFEP of the Federal Ministry of Health, with the assistance of The Carter Centre has set 2015 as the year to eliminate the disease in Nigeria. Even though a number of investigators [11-14] have carried out studies in different parts of the country, there is need to collate epidemiological data because most areas of the country have not yet received attention

The main objective of this study was to investigate the status of this infection amongst the Yakurr people of Cross River State, Nigeria, prior to the launch of the lymphatic filariasis elimination programme in Cross River Basin, where annual mass administration of ivermectin has been going on for several years through Community Directed Treatment.

## Methods

### Study area

A preliminary survey of lymphatic filariasis caused by *W. bancrofti* was carried out among the Yakurr people of Cross River State, Nigeria. This area is situated between latitude 5°40^′^ and 6°N and longitude 7°55^′^ and 8°33^′^ E. The communities randomly selected for the investigation included; Idomi, Ijom, Lekpankom and Ntankpo. They are The African Programme for Onchocerciasis Control (APOC) Community Directed Treatment with Ivermectin (CDTi) communities, and have been receiving treatment since 1995. They have a therapeutic coverage of over 80%. The Yakurr people are mainly farmers cultivating mainly yams, cassava and rice. This area is located in the tropical rainforest belt with high annual rainfall, high relative humidity and high mean annual temperature [15].

### Ethical consideration

Permission to conduct this survey was obtained from The Centre for Clinical Governance Research and Training of the Ministry of Health, Cross River State, Nigeria, before the commencement of the study. Consent was also obtained from the Paramount Ruler and the respective Community Heads of the Yakurr Local Government Area where investigation was carried out. They were informed of the nature, scope and purpose of the study. Peripheral night blood collection from the subjects and inconveniences of pains on pricking the finger were explained. Participation of individuals in these communities was voluntary.

### Sample collection

The sample for the present survey was drawn from household selected through systematic sampling with random start, selecting every alternate household. Each selected household was considered as a sampling unit as previously described [14]. Participants from the various communities were mobilized by the respective Community Heads to gather in their respective Community Health centre between 2300 and 0300 hours. Information about age, sex, history of previous treatment with ivermectin were obtained from each individual and recorded on a standard form. A total of 785 finger prick blood samples were collected from the four communities between March and August, 2009.

### Parasitological examination

Giemsa stained thick blood smear was used for the identification of microfilariae using a binocular microscope. Microfilariae of *W. bancrofti* were identified based on specific morphological features and sizes as described by Cheesbrough [16]. They were counted in each for the infected morphological density and recorded on the standard individual forms. The keys of Cheesbrough [16] were used to identify and distinguish microfilariae of *W. bancrofti* from microfilariae of other filarial worms, *Mansonella perstans* and *Loa loa*

### Clinical examination

Trained medical personnel recruited for this study examined all the 785 persons for clinical manifestation of lymphatic filariasis. All males and females were examined for signs of the limb and breast elephantiasis. Males had their genitals examined for hydrocoele and scrotal elephantiasis. Clinical manifestations were staged as described [17].

### Statistical analysis

The differences in the distribution of lymphatic filariasis among communities and age groups were determined using the analysis of variance (ANOVA). The prevalence of infection between sexes, and age was determined by chi-square test, while student ‘t’ test determined differences in density. All data were analyzed using SPSS for windows version 10.0 (SPSS Inc Chicago, IL).

## Results

A total of 785 Yakurr people of Cross River State were examined for lymphatic filariasis due to *W. bancrofti*. Sampling coverage ranged from 11.1% to 18.4% between the communities. It was observed that 48 (6.1%) out of the 785 persons were infected (Table 1). The prevalence of infection showed significant variation (P < 0.05) among the communities ranging from 4.1% in Idomi to 8.5% in Ntankpo (Table 1).

**Table 1:** **Prevalence (%) and clinical manifestation of lymphatic filariasis due to *W. bancrofti *by community**

**Community**	**Population**	**Population sampled (%)**	**Microfilaraemia positives**	**Lymphoedema Positives**
Idomi	1,204	221(18.4)	9 (4.1)	0 (0.0)
Ijom	1,311	151(11.5)	10 (6.6)	1 (0.7)
Lekpankom	1,584	224(14.1)	13 (5.8)	0 (0.0)
Ntankpo	1,699	189(11.1)	16 (8.5)	1 (0.5)
**Total**	**5,798**	**785(13.5)**	**48 (6.1)**	**2 (0.3)**

The prevalence of lymphatic filariasis by age is shown in Table 2. There was a gradual increase in the disease prevalence with age, reaching a peak in the 41 – 60 years old age group before the decline (Table 2). There was significant difference (P < 0.01) in the prevalence of lymphatic filariasis among the various age groups.

**Table 2:** **Prevalence (%) of *W. bancrofti *infection by age**

**Age range (Years)**	**No. of individuals examined**	**No (%) positive for microfilariae of LF**
1 – 20	36	9 (2.5)
21 – 40	184	16 (8.7)
41 – 60	157	19 (12.1)
61 – 80	79	4 (5.1)
**Total**	**785**	**48 (6.1)**

Of the 785 persons examined, 317 and 468 were males and females respectively, and 18 (5.7%) and 30 (6.4%) males and females were infected respectively (Table 3). Although more females were infected than males, there was no significant difference (P >0.05).

**Table 3:** **Prevalence (%) and mean density of *W. bancrofti *infection by community and sex**

**Community**	**Total number of individuals examined**		**Number (%) infected**		**Mean microfilarial density mf/50 μl**		**Total mf/ 50 μl**
	**Male**	**Female**	**Male**	**Female**	**Male**	**Female**	
Idomi	93	128	3 (3.2)	6 (4.7)	6.0	6.5	6.3
Ijom	64	87	4 (6.3)	6 (6.9)	6.5	6.3	6.4
Lekpankom	80	144	5 (6.3)	8 (5.6)	4.4	5.0	4.7
Ntankpo	80	109	6 (7.5)	10 (9.2)	4.0	5.3	4.8
**Total**	**317**	**468**	**18 (5.7)**	**30 (6.4)**	**5.2**	**5.8**	**5.6**

The microfilarial density indicated a mean overall value of 5.6 mf/50 μl of blood, (Table 3). There was no significant difference in the density of microfilaraemia between males and females (P > 0.05) Table 3. There was a significant variation (P < 0.01) in microfilarial density between the communities it ranged from 4.7mf/50 μl in Lekpankom to 6.4mf/50 μl in Ijom.

The only clinical manifestation of lymphatic filariasis due to *W. bancrofti* in the study population was lymphoedema (Table 1). Out of the 785 persons examined, 2 (0.3%) showed stage 2 lymphoedema of both legs. No other clinical manifestation were encountered.

## Discussions

This preliminary survey of lymphatic filariasis among 785 Yakurr people of Cross River State, Nigeria revealed an overall prevalence of 48 (6.1%). This is consistent with the results obtained by Udoidung et al. and Targema et al. [14,18] who reported 6.5% and 5.5% in Benue State and in some rural communities of the Lower Cross River Basin, Nigeria respectively. Higher prevalence was found in other studies of lymphatic filariasis due to *W. bancrofti* in Nigeria. Work by Okon et al. [12] reported 15.5% among Mbembe people of Cross River State, Anosike et al. [19] reported 16.9% among the Ezza people of Ebonyi State while Mbah and Njoku [20] observed 18.8% in Aguata Local Government Area of Anambra State. The lower prevalence in the present study could be due the current Government effort of CDTi project where ivermectin is being distributed in some communities in the Cross River State, including those of the Yakurr people. Ivermectin has the ability to eliminate microfilariae of other filarial parasites such as *M. perstans* and *Loa loa* including *W. bancrofti*[21]. The potential benefit of overlapping interventions have recently been highlighted elsewhere, and is an important consideration for determining the risk of lymphatic filariasis in Nigeria [22].

This study indicated a gradual increase in prevalence with increasing age range, thereafter there was a decline at the age range of 61 – 80 years. The prevalence observed among the 61 – 80 year age group may be due to resistance to new infection resulting from acquired immunity. However, a peak prevalence of 12.1% was recorded in the 41 – 60 year age group during this study. In a related study, Anosike [11] observed a gradual increase in prevalence with increasing age and a peak prevalence at age range of 40 – 49 years. In this study, the high prevalence observed between 21 – 60 years might be due to the fact that these age groups are involved in intense occupational activities such as farming and fishing. Typical Yakurr farmers spend three-quarters of the daytime in outdoor farming activities, hence they were exposed to more mosquito vectors in water pools prevailing in their cassava farms and rice fields.

The prevalence rate for males and females were 18 (5.7%) and 30 (6.4%) respectively. There was no statistically significant difference between both sexes. Both men and women engage equally in the activities that exposed them to the vectors. They were also exposed to the same living conditions. Most houses in the area had mud walls, thatched roofs, no ceiling; hence permitting movement of insects in and out as was earlier reported [14]. Also exposure to various breeding sites of the vectors due to their poor environmental and unhygienic conditions might have accounted for the equal prevalence of both males and females.

A mean microfilarial density of 5.6mf/50 μl of night peripheral blood was found among the infected population of Yakurr people of Cross River State, Nigeria. Okon et al. [12] found a mean density of 9.9mf/50 μl among the Mbembe people of Obubra Local Government Area of Cross River State. While Anosike [11] found a mean microfilarial density of 10.4mf per 20 mm^3^ of night peripheral blood. Microfilarial density is an important index in the epidemiology, treatment and control of human filariasis in endemic foci. The mean microfilarial density of 5.6 mf/50 μl obtained in this study is lower than the 9.9mf/50 μl and 9mf/50 μl obtained by [12,14] respectively. In spite of the low microfilarial density in the study area, there is a need for implementation of the various control measures in this locality. The low parasite density might also be due to the ongoing CDTi in the area, since the drug has an effect on *W. bancrofti*[21,23].

The only clinical manifestation of the lymphatic filariasis infection was lymphoedema found in 2 (0.3%) of the entire study population. This has been reported by several investigators as the cause of disability and disfigurement in endemic areas [24-26]. Hydrocele which is an important clinical manifestation of lymphatic filariasis was not observed in this study as was the case with previous investigations in the state [12,14]. The low prevalence of lymphoedema and absence of hydrocele might be due to human or parasite genetic differences or transmission related factors [27].

## Conclusions

This is the first documented report of lymphatic filariasis among the Yakurr people of Cross River State, Nigeria. This will thus enrich the epidemiological baseline data of the disease in Nigeria. The Government should intervene by expanding the distribution of albendazole in addition to the CDTi that is presently going on in the communities. Preventative chemotherapy and transmission control (PCT) with albendazole and ivermectin annually in populations at risk where lymphatic filariasis prevalence of more than or equal to 1% is the basis of lymphatic filariasis elimination programmes [28,29].

## Competing interests

We have no competing interest.

## Authors’ contributions

ICI, OOE, OKN, took part in the design and conception of the study. They also contributed in the writing of the manuscript. ASE, ESE, took part in the data collection, analysis and writing of the manuscript. All authors read and approved the final manuscript.
